# Parasitism of *Placobdelloides siamensis* (Oka, 1917) (Glossiphoniidae: Hirudinea) in Snail-eating Turtles, *Malayemys* spp., and the effects of host and aquatic environmental factors

**DOI:** 10.3897/BDJ.8.e57237

**Published:** 2020-10-26

**Authors:** Poramad Trivalairat, Krittiya Chiangkul, Watchariya Purivirojkul

**Affiliations:** 1 Animal Systematics and Ecology Speciality Research Unit, Department of Zoology, Faculty of Science, Kasetsart University, 50 Ngam Wong Wan Road, Chatuchak, Bangkok, 10900, Thailand Animal Systematics and Ecology Speciality Research Unit, Department of Zoology, Faculty of Science, Kasetsart University 50 Ngam Wong Wan Road, Chatuchak, Bangkok, 10900 Thailand

**Keywords:** Rhynchobdellida, Geoemydidae, *Malayemys*; aquatic environment, distribution, Thailand

## Abstract

The Siam Shield Leech, *Placobdelloides siamensis*, is a common leech found on Malayemys turtles in Thailand. Sixty Snail-eating Turtles (29 *Malayemys macrocephala* and 31 *M. subtrijuga*) were caught over twelve months (February 2017 – January 2018) to determine host characteristics (body size, weight and sex), parasitism (prevalence, intensity and density) and seasonal aquatic environmental factors (conductivity, nitrate nitrogen, dissolved oxygen, pH, salinity and total dissolved solids). There was no significant difference of infection rate between species and sex in both turtle species. Leech prevalence indicated that all turtle individuals were infected throughout year, while the infection rate was significantly higher in larger and heavier turtles mainly on the carapace with an average number of leech approximately 474.80 ± 331.38 individuals for individual host infection and 76.53 ± 20.27 individuals for infection per 100 g body weight. The high level of leech parasitism also caused a rot wound and shell hole which caused the host to die. Aquatic environmental factors did not influence the infection of leeches in both turtle species. Therefore, the factors that influenced the infection rate of *P. siamensis* were based on only host body size and weight without effect from season. In addition, this study also showed two new hosts, including *Cyclemys oldhamii* and *Heosemys grandis* and the widespread distribution from northern, north-eastern, western, central and southern Thailand were reported.

## Introduction

Leeches are widespread ectoparasites found in various habitats including terrestrial, marine and particularly freshwater environments (Sawyer 1986). *Placobdelloides* Sawyer, 1986 is a freshwater leech genus distributed globally in various hosts, such as water snails, fishes, amphibians, reptiles and mammals (Sawyer 1986, Tucker et al. 2005, Alam et al. 2008, Schulz et al. 2011, Tubtimon et al. 2014). The leeches in family Glossiphoniidae, including *Placobdelloides* leeches, exhibit a parental care behaviour, which is a unique behaviour that helps to increase the survival rate of their young (Kutschera 1992, Kutschera and Wirtz 2001, Bolotov et al. 2019). In Thailand, two *Placobdelloides* species are reported: first is *Placobdelloides siamensis* (Oka, 1917) from the Black Marsh Turtle (*Siebenrockiella crassicollis* (Gray, 1831)), Khorat Snail-eating Turtle (*M. Khoratensis* Ihlow et al., 2016), Malayan Snail-eating Turtle (*M. Macrocephala* (Gray, 1859)), Mekong Snail-eating Turtle (*Malayemys subtrijuga* (Schlegel and Müller, 1845)), Southeast Asian Box Turtle (*Cuora amboinensis* (Daudin, 1802)) and the Yellow-headed Temple Turtle (*Heosemys annandalii* (Boulenger, 1903)); and the recently-described species is *P. sirikanchanae* Trivalairat, Chiangkul and Purivirojkul, 2019 from Asian Leaf Turtle (*Cyclemys dentata* (Gray, 1831)) and the Dark-bellied Leaf Turtle (*C. enigmatica* Fritz et al., 2008) (Chiangkul et al. 2018, Trivalairat et al. 2019). Many leeches are haematophagous and have anti-coagulants in their saliva, which can cause haemorrhage and sometimes decrease blood volume and nutrients of the hosts (Bragg et al. 1989, Rigbi et al. 1987, Tiberti and Gentilli 2010). Moreover, the blood-feeding leeches can acquire blood-borne parasites and act as a vector to a future host (Kikuchi and Fukatsu 2002, Mihalca et al. 2002). Consequently, the leeches may increase mortality and risk of disease to hosts, especially wetland aquatic biomonitoring vertebrate species.

The Malayan Snail-eating Turtle (*Malayemys macrocephala* (Gray, 1859)) and Mekong Snail-eating Turtle (*M. subtrijuga*) are aquatic freshwater turtles distributed mainly in Thailand (Schlegel and Müller 1845, Gray 1859, Das 2010). They live near slow-flowing freshwater or shallow lacustrine freshwater bodies that are covered with dense vegetation, such as swamps, canals, ditches and flood fields (Ernst et al. 1997, Bonin et al. 2006, Satun inland aquaculture research and development center 2009). In addition, these turtles are important for biological control by consuming the invasive Golden Apple Snail (*Pomacea canaliculata* (Lamarck, 1819)) (Lamarck 1819, Carlsson et al. 2004). In Thailand, people usually release turtles, especially Snail-eating Turtles, into canals or ponds for merit (Faculty of Veterinary Science, Chulalongkorn University 2005, Asian Herp Blogs 2011, Chiangkul et al. 2018). However, as found in this study, the Siamese Shield Leech is observed in both species of Snail-eating Turtles. This means that the release of turtles potentially causes an occasional dispersion of the leech into natural habitats, which involves infecting and worsening the health of other hosts.

## Material and methods

### Specimen Collection and Host Measurements

Five individuals of the Snail-eating Turtles (*Malayemys macrocephala* or *M. subtrijuga*) were randomly captured by hand on the same day each month (15^th^ day, during night) for 12 months continuously (February 2017 through to January 2018) from ponds in Kasetsart University, Bangkok, Thailand (13°50’53.6”N 100°33’47.3”E). All of the captured turtles were provided to the laboratory in the Department of Zoology, Faculty of Science, Kasetsart University to be examined for weight (g), carapace length (cm) and sex. Weight and measurement were recorded using regularly calibrated digital scales (Teneca digital medical scales) and Vernier calliper (nearest 0.1 mm), respectively. Sex identification was identified from tail and cloaca following Keithmaleesatti 2008. Then, the total number of mature leeches (excluding juveniles and eggs) were counted and removed from the host’s outer area using forceps. Leeches were identified following Chiangkul et al. 2018 and stored in 70% alcohol. All turtles were released to their capture site when finished.

In addition, to avoid forcefully removing the leeches and causing damage, each turtle was kept moist, because, in this study, some turtles were left in a tank without water overnight, causing almost all of the leeches on the carapace and plastron to shrink and die, except for the leeches that moved to the head, axillar, groin and caudal regions where there was more moisture than found on the shells. As a result, the turtles were always kept moist by keeping them in a water tank to avoid biasing the leech infection.

### Water Analysis

For 12 continuous months (February 2017 through to January 2018), after collecting the turtles, the water at the sites and depths of each turtle capture were measured for environmental factors such as conductivity (µs/cm), nitrate nitrogen (NO_3_-N) (mg/l), optical dissolved oxygen (ODO) (mg/l), pH, salinity (ppt), temperature (ᴼC) and total dissolved solids (TDS) (mg/l), using a YSI EXO multiparameter instrument (YSI Incorporated, Yellow Springs, Ohio, USA) to investigate the relationship between leech parasitism and any seasonal environmental factors.

### Survey Sampling

Geoemydidae turtle species were surveyed and captured by hand from the natural habitats and captive sites (temples), including markets within Thailand during February 2017 through to June 2018 to investigate a host-specific relationship and distribution of *P. siamensis*. The number of leech and effects from leech infestation on each turtle were immediately recorded in fields and turtles were released back to their capture site when recordings were complete.

### Statistical Analysis

Prevalence (the percentage of hosts infected with at least one leech), mean intensity (the average number of leeches per infected host) and mean density (the average number of leeches per 100 g body weight of infected host) were determined throughout the year. Prevalence and mean intensity were performed following Bush et al. 1997, while density was calculated to minimise bias between weight and body size variations.

\begin{varwidth}{50in}
        \begin{equation*}
            Prevalence\space (\%) = {(Total\space infected\space hosts)*100 \over Total\space hosts}
        \end{equation*}
    \end{varwidth}

\begin{varwidth}{50in}
        \begin{equation*}
            Intensity\space (individuals) = {Total\space numbers\space of\space leech \over Total\space infected\space hosts}
        \end{equation*}
    \end{varwidth}

\begin{varwidth}{50in}
        \begin{equation*}
            Density\space (individuals/ 100g)= {(Number\space of\space leech)*100 \over Turtle\space weight}
        \end{equation*}
    \end{varwidth}

The IBM SPSS Statistics software package (SPSS Inc.; Chicago, IL, USA) was used to analyse the number of leeches, carapace length and weight with a 5% type I error risk. Leech loads, numbers of leech, intensity and density, were not normally distributed, so non-parametric tests were used to compare leech load amongst population and other variables. The mean intensity (individuals) and mean density (individuals/100g) of *P. siamensis* from *M. macrocephala* and *M. subtrijuga*, including differences between sexes in both species, were analysed using the Mann-Whitney U test. Spearman's rank correlation was used to examined the relationships between leech loads (number of leech) and host characteristics (weight and carapace length) and mean density during the 12 months (February 2017 – January 2018) and seven variables of water analysis: conductivity (µs/cm), nitrate nitrogen (NO_3_-N) (mg/l), optical dissolved oxygen (ODO) (mg/l), pH, salinity (ppt), specific conductance (SPC) (µs/cm) and total dissolved solids (TDS) (mg/l). The preference area infection on hosts (carapace, head and axilla, groin and tail and plastron) and mean density in each month were analysed using one-way ANOVA.

## Results

### Turtle Body Size

Two species of turtle, *Malayemys macrocephala* and *M. subtrijuga*, were captured in Kasetsart University. A total of 29 individuals (21 females and 8 males) of *M. macrocephala* were captured; they had a mean weight of 709.14 ± 462.92 g (min-max: 80-1700 g) (812.38 ± 457.19 g (min-max: 80-1700 g) for females and 438.13 ± 462.92 g (min-max: 150-1300 g) for males) and carapace length of 16.20 ± 4.71 cm (min-max: 7.8-23.0 cm) (17.20 ± 4.38 cm (min-max: 7.8-23.0) for females and 13.60 ± 4.85 cm (min-max: 9.5-21.5 cm) for males). A total of 31 individuals (21 females and 10 males) of *M. subtrijuga* were captured; they had a mean weight of 572.24 ± 437.04 g (min-max: 19-1500 g) (699.02 ± 470.81 g (min-max: 19-1500 g) for females and 306.00 ± 166.88 g (min-max: 38-779 g) for males) and carapace length of 15.21 ± 4.53 cm (min-max: 4.8-23.0 cm) (16.35 ± 4.83 cm (min-max: 4.8-23.0 cm) for females and 12.82 ± 2.66 cm (min-max: 9.1-18.8 cm) for males). These results indicated that *M. macrocephala* was larger and heavier than *M. subtrijuga* and that females of both species were larger and heavier than males.

### Preference between Species

**Figure 1.**
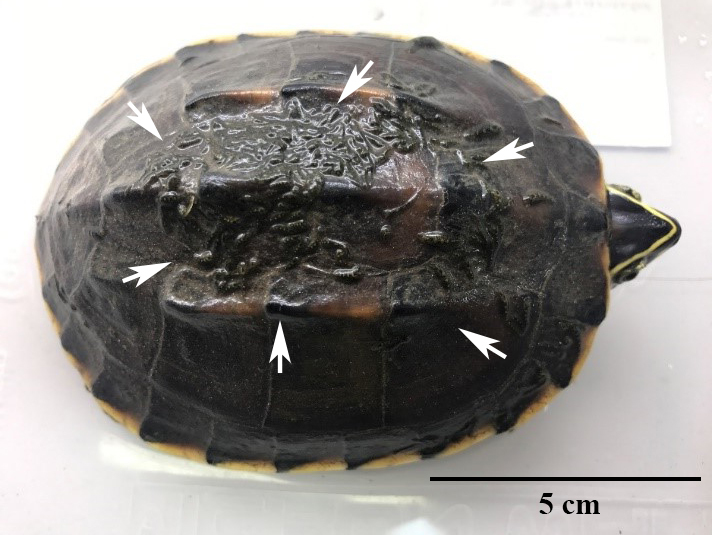
*Placobdelloides siamensis* on *Malayemys subtrijuga* carapaces.

**Figure 2.**
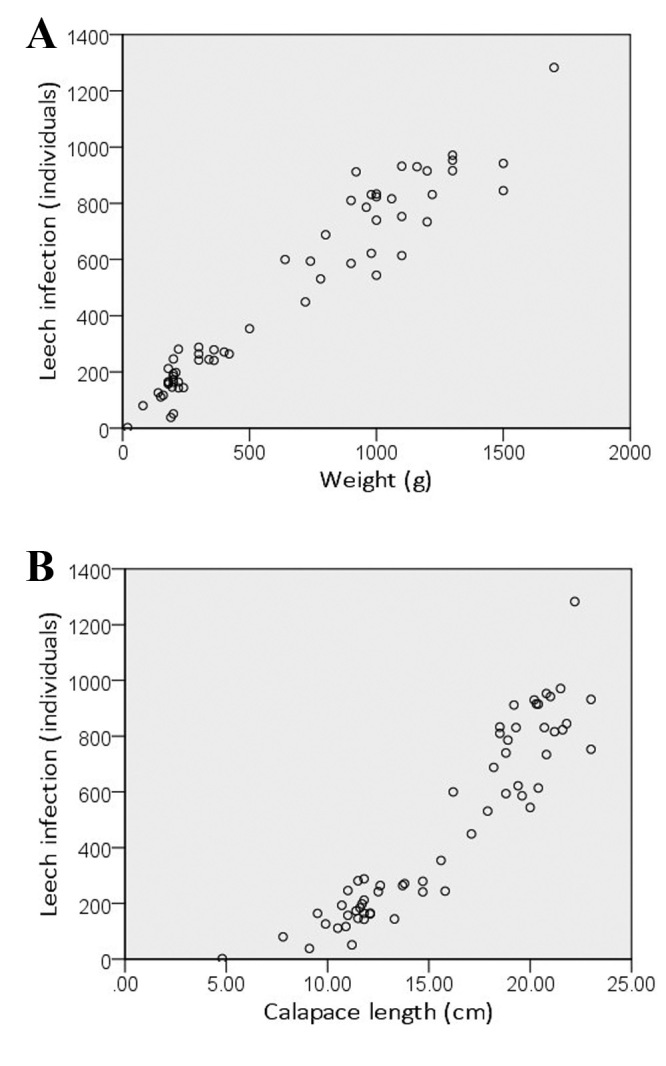
Leech infection vs. turtle weight (A) and vs. carapace length scatterplots (B)

The captured turtles were parasitised by a single species of leech, *Placobdelloides siamensis*, totalling 28,488 individuals from 60 host specimens (Fig. 1). Five mature leech specimens (ZRC.ANN.0435 to 0439) from each turtle in the first month were deposited in the Zoological Reference Collection (ZRC) of the Lee Kong Chian Natural History Museum (LKCNHM), National University of Singapore, Singapore and others series of leech collection from each turtle (60 catalogue numbers, ZMKU-ANN-SER-0001 to 0059) were deposited in the Zoological Museum, Department of Zoology, Faculty of Science, Kasetsart University, Bangkok, Thailand (ZMKU). The leech infection in both turtle species was found to be distributed with the mean number of leech 538.24 ± 356.26 individuals for *M. macrocephala* (609.43 ± 343.19 individuals for females and 351.38 ± 340.66 individuals for males) and 415.45 ± 299.96 individuals for *M. subtrijuga* (493.62 ± 326.36 individuals for females and 242.30 ± 143.62 individuals for males) (Table 1). The leech infection in both species and sex increased significantly with increasing weight (*r* = 0.926, *p* = 0.000; *r* = 0.843, *p* = 0.009 for females and males of *M. macrocephala*) (*r* = 0.928, *p* = 0.000; *r* = 0.908, *p* = 0.000 for females and males of *M. subtrijuga*) and body size (carapace length) (*r* = 0.830, *p* = 0.000; *r* = 0.766, *p* = 0.027 for females and males of *M. macrocephala*) (*r* = 0.925, *p* = 0.000; *r* = 0.793, *p* = 0.006 for females and males of *M. subtrijuga*) (Table 2) (Fig. 2). Moreover, the turtle weight increased significantly with increasing body size (*r* = 0.901, *p* = 0.000 for females of *M. macrocephala*) (*r* = 0.960, *p* = 0.000; *r* = 0.941, *r* = 0.000 for females and males of *M. subtrijuga*), except males *M. macrocephala* (*r* = 0.576, *p* = 0.135) . Hence, the females individuals in both species have a tendency to be infected by leeches more than males from larger carapace lengths and weights.

*Placobdelloides siamensis* demonstrated no differences of intensity between *M. macrocephala* and *M. subtrijuga* (*u* = 1.448, *p* = 0.119), as well as no differences of infection between females and males in *M. macrocephala* (*u* = 115.0, *p* = 0.070) and *M. subtrijuga* (*u* = 144.0, *p* = 0.053) (Table 1).

The mean density in both species indicated approximately 78.86 ± 14.10 individuals/100g for *M. macrocephala* (76.44 ± 11.72 individuals/100g for females and 85.21 ± 18.43 individuals/100g for males) and 74.35 ± 24.75 individuals/100g for *M. subtrijuga* (78.35 ± 26.15 individuals/100g for females and 78.35 ± 22.27 individuals/100g for males) (Table 1). These densities also demonstrated no differences between *M. macrocephala* and *M. subtrijuga* (*u* = 409.0, *p* = 0.275), as well as no differences of infection between females and males in *M. macrocephala* (*u* = 55.5, *p* = 0.084) and *M. subtrijuga* (*u* = 68.0, *p* = 0.062) (Table 1). These results indicated *P. siamensis* had no host specific preference between these two turtle species and could be treated as similar populations.

### Infection Site Preference

The external body surface of both species were infected mostly on the carapace (311.00 ± 208.99 individuals (57.78%) for *M. macrocephala*) (241.94 ± 181.22 individuals (56.57%) for *M. subtrijuga*), followed by: head and axilla (93.24 ± 72.62 individuals, 17.32%), groin and caudal (64.11 ± 11.91 individuals, 16.56%) and plastron (34.07 ± 6.33 individuals, 8.33%), respectively, for *M. macrocephala*; head and axilla (69.65 ± 59.43 individuals, 20.06%), groin and caudal (70.52 ± 58.90 individuals, 16.46%) and plastron (30.45 ± 25.73 individuals, 6.91%), respectively, for *M. subtrijuga* (Table 3).

### Prevalence and Density

**Figure 3.**
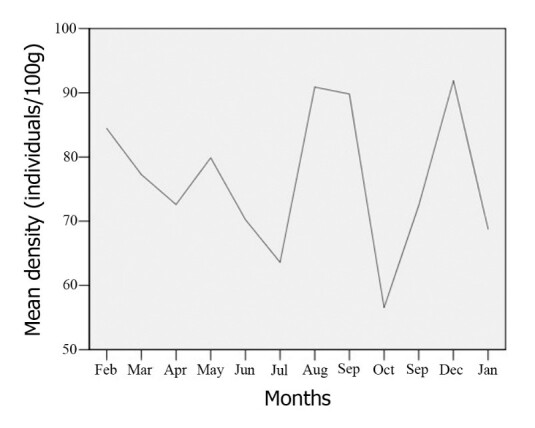
Mean density (individuals/100g) of *Placobdelloides siamensis* on Malayemys turtles by month in Kasetsart University, Bangkok, Thailand from February 2017 to January 2018.

A high level of infection was found with 100% of turtles infected (including a hatchling) throughout the year in these populations. The mean density through the year resulted in 76.53 ± 20.27 individuals/100g (Fig. 3). In addition, the mean density demonstrated no difference of infection in each month (f = 1.754, p = 0.90).

### Environmental Factors

Leech density on both turtle species (*M. macrocephara* and *M. subtrijuga*) was not affected by conductivity (*r* = -0.118, *p* = 0.370), nitrate nitrogen (NO_3_-N) (*r* = 0.017, *p* = 0.898), optical dissolved oxygen (ODO) (*r* = -0.173, *p* = 0.186), pH (*r* = 0.071, *p* = 0.591), salinity (*r* = -0.106, *p* = 0.422), temperature (*r* = 0.091, *p* = 0.488) or total dissolved solid (TDS) (*r* = -0.117, *p* = 0.373) throughout the year (February 2017 to January 2018) (Table 4).

### Distribution

**Figure 4.**
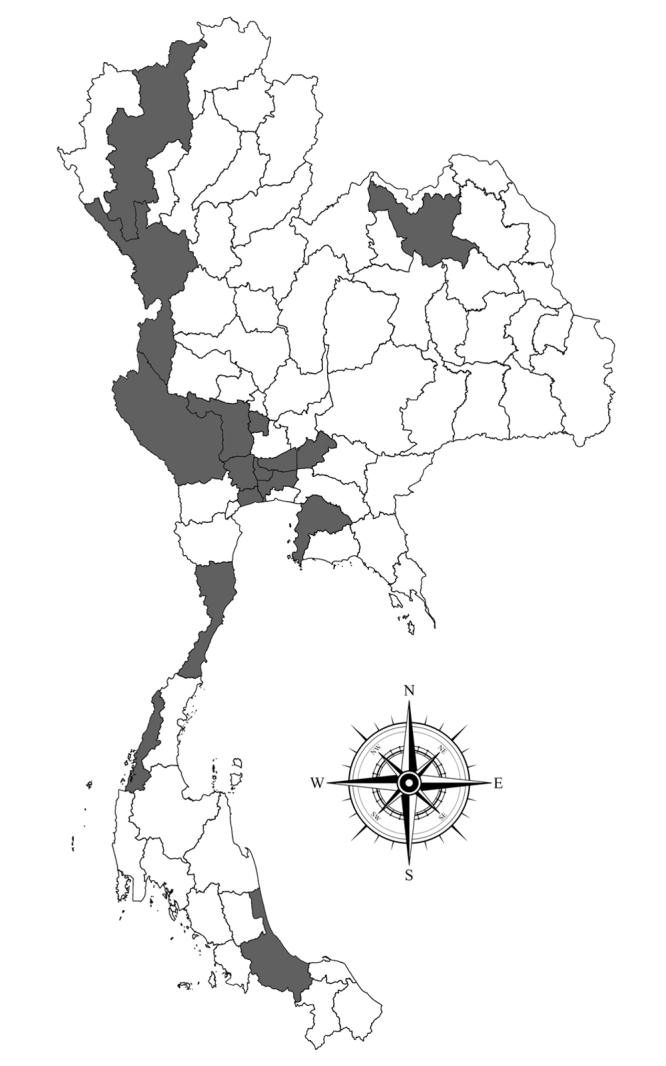
Distribution of *Placobdelloides siamensis* in Thailand.

**Figure 5.**
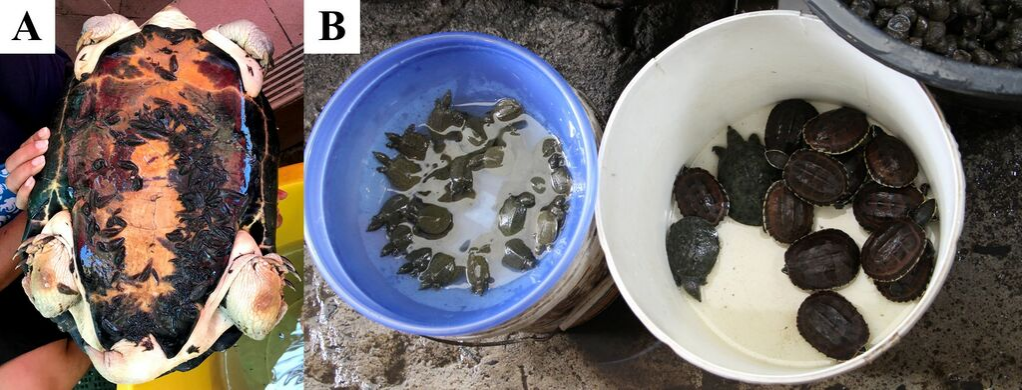
Illegal turtle trading for merit releasing in Thailand: (A) *Hieremys annandalii* with massive parasitism of *Placobdelloides siamensis* from Soi Thong Temple, Bang Sue, Bangkok; and (B) *Malayemys subtrijuga* from Warorot Market, Mueang, Chiangmai.

Altogether, eight species of Geoemydidae turtle from 16 provinces in Thailand were found infected by *P. siamensis* as follows (Figs 4, 5) (Table 5) (Suppl. material 1): *Malayemys macrocephala* from Ang Thong, Bangkok, Chiangmai, Nakhon Pathom, Nonthaburi, Pathumthani and Suphan Buri; *M. subtrijuga* from Ang Thong, Bangkok, Nakhon Nayok, Nonthaburi and Suphan Buri; *M. khoratensis* from Udon Thani; *Cuora amboinensis* from Bangkok, Chonburi, Kanchanaburi, Ranong and Songkhla; *Cyclemys oldhamii* (Gray, 1836) from Prachuap Khiri Khan and Tak; *Heosemys grandis* (Gray, 1860) from Chonburi; *Hieremys annandalii* from Bangkok, Chonburi, Kanchanaburi, Ranong and Samut Sakhon; *Siebenrockiella crassicollis* from Bangkok.

The invasive turtle species, *Trachemys scripta elegans* (Thunberg in Schoepff, 1792), was found from Bangkok and Chonburi without leech infection.

### Symptoms of Infection

**Figure 6.**
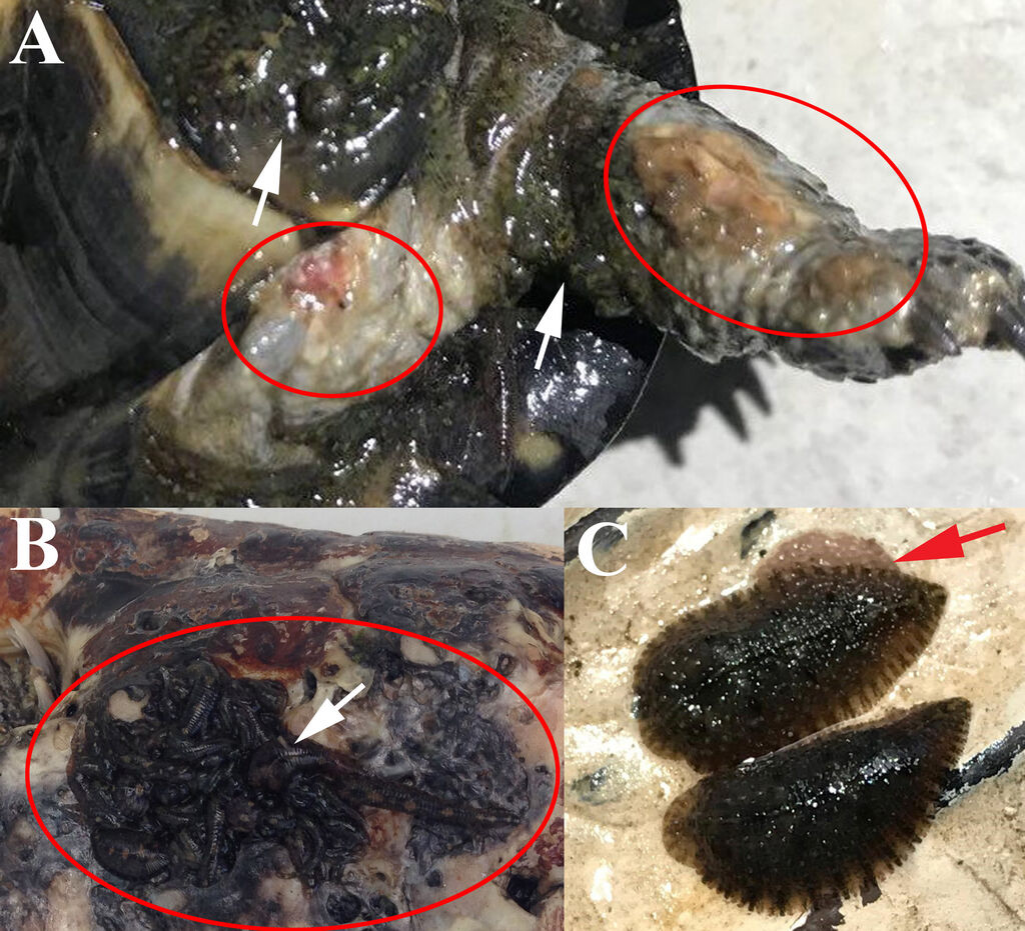
Symptoms of *Placobdelloides siamensis* infection (white arrows). (A) Epidermal lesion on forelimb and axilla (red circles) of *Siebenrockiella crassicollis*; (B) Penetration under keratinised scute on plastron (red circles) of *Malayemys subtrijuga*; (C) Egg deposition on carapace (red arrow) of *M. subtrijuga*.

This is the first record of leech infested turtles from surveying in Thailand. The aggregated infection of *P. siamensis* could cause peeling shells, shell holes, haemorrhage or lesions on epidermal tissues towards the *S. crassicollis* from Bangkok from tissue consumption (Fig. 6A). This leech also penetrated under the keratinised scute on the plastron and bone tissue (shell) through the soft tissue to consume the tissue and blood meals of *M. subtrijuga* from Nonthaburi (Fig. 6B). Additionally, it occasionally deposited and raised its eggs (approximately 200-400 eggs/clutch) on the carapace surface (Fig. 6C).

## Discussion

### Host comparisons

Generally, adult *Malayemys macrocephala* are usually larger than *M. subtrijuga* and the females in both species are larger than the males (Ernst et al. 1997, Bonin et al. 2006, Satun inland aquaculture research and development center 2009, Das 2010). The findings of this study from Kasetsart University, Bangkok, Thailand, were in agreement with this; *M. macrocephala* showed a mean weight and carapace length larger than *M. subtrijuga* and females in both species were found to have approximately 30% larger carapace lengths and twice the weight of males. This sexual dimorphism may be an adaptation that allowed smaller males to be ready for mating, while allowing larger females to gather more nutrients and energy to produce more offspring (Berry and Shine 1980, Shine 1988).

### Parasitic-Host Relationship

The populations of *M. macrocephala* and *M. subtrijuga*, from Kasetsart University, Bangkok, Thailand, were determined to be the hosts of a single observed leech, *Placobdelloides siamensis*. *P. siamensis* was mostly concentrated on the carapace region in both species. The colonisation of leech on the carapace region might be an adaptation to rest after a blood meal, because this region was influenced less from turtle motions, whereas, head, axilla, groin and caudal regions were epidermal tissues from which leeches could have a blood meal and were also susceptible for leech parasitism from benthos (Ernst et al. 1997, Graham et al. 1997, Ryan and Lamber 2005, Bonin et al. 2006, McCoy et al. 2007). However, these regions were frequent locomotion parts that might disturb leech attaching. Including the plastron region, scratching from the ground also disturbed the attachment of leeches. Consequently, the leech infection was discovered to be mostly on carapace areas thus avoiding the disturbances from turtle activities in other regions.

Furthermore, the results demonstrated that every single Malayemys turtle in Kasetsart University was infected by *P. siamensis* throughout the year (February 2017 through to January 2018) and infection was even found on a young hatchling. The leech infection increased relative to the host body size and weight. As seen in most animals, body size is positively correlated to weight. In addition, this leech is a blood-feeding ectoparasite that attaches, including reproducing, to the outer parts of the hosts longer than the temporary buffalo leeches which leave the host after sufficient infestation has occurred (Southerland 1986a, Southerland 1986b, Goater 2000, McCoy et al. 2007, Readel et al. 2008, Peig and J.Green 2010). Therefore, the increasing host surface also provides more living areas for attachment and a greater blood resource for feeding.

### Environmental Effects

The seven analysed water variables (conductivity, nitrate nitrogen (NO_3_-N), optical dissolved oxygen (ODO), pH, salinity, temperature and total dissolved solid (TDS)) are essential for some aquatic organisms for balance, water balance support, nutrients and respiration (Mann 1962, Davies 1991, Bush et al. 1997, American Public Health Association et al. 1999, Wetzel 2001, Hayashi 2004, Iyasele et al. 2015, Desai and Rao 2016, Mueller and Helsel 2016). However, in this study, the seven variables were not significantly related to leech intensity throughout the survey period. Accordingly, the leech intensity was not related to conductivity, NO_3_-N, ODO, pH, salinity, temperature and TDS in each season.

### Distribution in Thailand

Although *Siebenrockiella crassicollis* is described as the original host of *P. siamensis* from Thailand, it is commonly found in *M. macrocephala* and *M. subtrijuga*, *M. khoratensis*, *Cuora amboinensis* and *Hieremys annandalii* (Annandale 1925, Sawyer 1986, Chiangkul et al. 2018). However, this study demonstrated the first record of *P. siamensis* from *Cyclemys oldhamii* and *Heosemys grandis*. In addition, this was the first distribution record of *P. siamensis* from the northern region (Chiangmai), western regions (Kanchanaburi and Tak), central regions (Ang Thong, Nakhon Nayok, Nakhon Pathom, Nonthaburi, Prachuap Khiri Khan, Pathumthani, Samut Sakhon and Suphan Buri) and southern regions (Ranong and Songkhla). Therefore, this leech had been shown to feed mostly on Geoemydidae turtles, as mentioned above and tended to spread throughout the Thailand area following its host distribution.

### Effects of Infection

*Placobdelloides siamensis* is a jawless leech (Rhynchobdellida) which uses a proboscis to obtain a blood meal by penetrating epidermal tissues under scales or bony tissues of turtle shells (Mann 1962, Siddall and Gaffney 2004). The chronic infection by a concentrated leeches colony damaged tissues due to direct penetration and also caused a wound on the epidermal tissues or shell holes. In addition, the higher leech parasitism also harm the turtle health from anaemia and malnutrition, including haemoparasite transmission, which can sometimes kill the turtles (Rigbi et al. 1987, Bragg et al. 1989, Brooks et al. 1990, Kikuchi and Fukatsu 2002, Tiberti and Gentilli 2010, Dvorakova et al. 2014). Occasionally, both the turtle species in the wild take aerial-basks to reduce leech loads by exposing the parasite to desiccation (Ernst 1971, McAuliffe 1977, Koffler et al. 1978). Consequently, the chronic infection of concentrated leech colonies could significantly effect turtle hosts, ultimately causing their death.

## Supplementary Material

461BE70A-EF8D-556A-9BC3-354898D22692 10.3897/BDJ.8.e57237.suppl1 Supplementary material 1 Recorded Specimen Data Data type Specimen collecting data Brief description Recorded data of collected Geoemydidae turtles from natural habitats (NH), captive site (CS) and markets (MA) in Thailand during February 2017 through to June 2018. All specimens were collected manually by Poramad Trivalairat, except specimens from markets. File: oo_458210.docx https://binary.pensoft.net/file/458210 Poramad Trivalairat.

## Figures and Tables

**Table 1.** The Mann-Whitney U test of leech intensity and density found on *Malayemys macrocephala* and *M. subtrijuga*, and between the sexes of both species.

**Variables**	**Intensity**			**Density**		
	**Mean**	***u***	***p***	**Mean**	***u***	***p***
* Malayemys *						
*macrocephala* (*n* = 29)	538.24 ± 356.26	369.5	0.119	78.86 ± 14.10	409.0	0.275
*subtrijuga* (*n* = 31)	415.45 ± 299.96			74.35 ± 24.75		
*M. macrocephala*						
Female (*n* = 21)	609.43 ± 343.19	115.0	0.070	76.44 ± 11.72	55.5	0.084
Male (*n* = 8)	351.38 ± 340.66			85.21 ± 18.43		
*M. subtrijuga*						
Female (*n* = 21)	493.62 ± 326.36	144.0	0.053	78.35 ± 26.15	68.0	0.062
Male (*n* = 10)	242.30 ± 143.62			78.35 ± 22.27		
Total	47480 ± 331.38			76.53 ± 20.27		

**Table 2.** Spearman's correlation (*r*) and *p* – values (*p*) of model variables: number of leeches, weight (g) and carapace length (cm) in both female (F) and male (M) of *Malayemys macrocephala* and *M. subtrijuga*.

**Characteristics**	***M. macrocephala***				***M. subtrijuga***			
	**F (*n* = 21)**		**M (*n* = 8)**		**F (*n* = 21)**		**M (*n* = 10)**	
	***r***	***p***	***r***	***p***	***r***	***p***	***r***	***p***
Number of leech								
Weight	0.926	0.000	0.843	0.009	0.928	0.000	0.908	0.000
Carapace length	0.830	0.000	0.766	0.027	0.925	0.000	0.793	0.006
Carapace length								
Weight	0.901	0.000	0.576	0.135	0.960	0.000	0.941	0.000

**Table 3.** One-way ANOVA resulted in the source of leech infected variation on the body surface of *Malayemys macrocephala* and *M. subtrijuga*: C = carapace region; HA = Head and axilla; P = plastron; GT = groin and tail.

**Variables**	**Number of leech in each site**				***f***	***p***
	**C**	**HA**	**P**	**GT**		
*M. macrocephala* (*n* = 29)	311.00 ± 208.99	93.24 ± 72.62	34.07 ± 6.33	64.11 ± 11.91	30.627	0.000
*M. subtrijuga* (*n* = 31)	241.94 ± 181.22	69.65 ± 59.43	30.45 ± 25.73	70.52 ± 58.90	27.283	0.000

**Table 4.** Spearman's correlation (*r*) and *p* – values (*p*) of model variables throughout the year (February 2017 to January 2018): leech density on turtles (*Malayemys macrocephala* and *M. subtrijuga*), conductivity (µs/cm), nitrate nitrogen (NO_3_-N) (mg/l), optical dissolved oxygen (ODO) (mg/l), pH, salinity (ppt), Temperature (°C) and total dissolved solid (TDS) (mg/l).

**Parameters**	**Mean**	***r***	***p***
Mean density			
1. Conductivity (µs/cm)	369.12 ± 289.93	-0.118	0.370
2. NO_3_-N (mg/l)	1.50 ± 1.38	0.017	0.898
3. ODO (mg/l)	3.80 ± 1.52	-0.173	0.186
4. pH	7.38 ± 0.26	0.071	0.591
5. Salinity (ppt)	0.17 ± 0.14	-0.106	0.422
6. Temperature (ᴼC)	28.18 ± 1.96	0.091	0.488
7. TDS (mg/l)	230.17 ± 184.15	-0.117	0.373

**Table 5.** The prevalence (%) and mean intensity (individuals) of Geoemydidae turtles in Thailand.

**Species**	**Number (*n*)**		**Prevalence (%)**	**Mean intensity** **(individuals)**
	**Infected**	**Examined**		
*Malayemys macrocephala*	55	55	100.0	96.96 ± 118.74
*Malayemys subtrijuga*	64	71	90.1	98.35 ± 159.05
*Malayemys khoratensis*	1	1	100.0	29
*Cuora amboinensis*	10	53	18.9	0.47 ± 1.51
*Cyclemys oldhamii*	2	2	100.0	14.50 ± 19.09
*Heosemys grandis*	1	3	33.3	2.33 ± 4.04
*Hieremys annandalii*	12	22	54.5	425.77 ± 538.46
*Siebenrockiella crassicollis*	3	3	100.0	8.67 ± 7.64
*Trachemys scripta elegans*	0	12	0	0
