# Supplementary material for: Parasitism of Placobdelloides siamensis (Oka, 1917) (Glossiphoniidae: Hirudinea) in Snail-eating Turtles, Malayemys spp., and the effects of host and aquatic environmental factors
Source: Biodivers Data J. 2020 Oct 26;8:e57237. doi: 10.3897/BDJ.8.e57237 (PMC7606437; doi:10.3897/BDJ.8.e57237)
Supplement: Supplementary material 1 — Recorded Specimen Data [file bdj-08-e57237-s001.docx]

Supplementary Material 1 Recorded data of collected geoemydidae turtles from natural habitats (NH), captive site (CS), and markets (MA) in Thailand during February 2017 through June 2018. All specimens were collected with hands by Poramad Trivalairat except specimens from markets.

| No. | Date | Species | Collected sites | Coordinates | Type sites |
| --- | --- | --- | --- | --- | --- |
| 1 | 15 February 2017 | *Malayemys subtrijuga* | Kasetsart University, Bangkhen, Bangkok | 13°50’53.6”N, 100°33’47.3”E | NH |
| 2 | 15 February 2017 | *Malayemys subtrijuga* | Kasetsart University, Bangkhen, Bangkok | 13°50’53.6”N, 100°33’47.3”E | NH |
| 3 | 15 February 2017 | *Malayemys macrocephala* | Kasetsart University, Bangkhen, Bangkok | 13°50’53.6”N, 100°33’47.3”E | NH |
| 4 | 15 February 2017 | *Malayemys macrocephala* | Kasetsart University, Bangkhen, Bangkok | 13°50’53.6”N, 100°33’47.3”E | NH |
| 5 | 15 February 2017 | *Malayemys macrocephala* | Kasetsart University, Bangkhen, Bangkok | 13°50’53.6”N, 100°33’47.3”E | NH |
| 6 | 16 February 2017 | *Trachemys scripta elegans* | Dusit Zoo, Dusit, Bangkok | 13°46'23.2"N, 100°31'02.5"E | NH |
| 7 | 16 February 2017 | *Trachemys scripta elegans* | Dusit Zoo, Dusit, Bangkok | 13°46'23.2"N, 100°31'02.5"E | NH |
| 8 | 16 February 2017 | *Trachemys scripta elegans* | Dusit Zoo, Dusit, Bangkok | 13°46'23.2"N, 100°31'02.5"E | NH |
| 9 | 16 February 2017 | *Trachemys scripta elegans* | Dusit Zoo, Dusit, Bangkok | 13°46'23.2"N, 100°31'02.5"E | NH |
| 10 | 16 February 2017 | *Trachemys scripta elegans* | Dusit Zoo, Dusit, Bangkok | 13°46'23.2"N, 100°31'02.5"E | NH |
| 11 | 16 February 2017 | *Trachemys scripta elegans* | Dusit Zoo, Dusit, Bangkok | 13°46'23.2"N, 100°31'02.5"E | NH |
| 12 | 16 February 2017 | *Trachemys scripta elegans* | Dusit Zoo, Dusit, Bangkok | 13°46'23.2"N, 100°31'02.5"E | NH |
| 13 | 16 February 2017 | *Trachemys scripta elegans* | Dusit Zoo, Dusit, Bangkok | 13°46'23.2"N, 100°31'02.5"E | NH |
| 14 | 23 February 2017 | *Malayemys macrocephala* | Mueang Nonthaburi Market, Mueang, Nonthaburi | 13°50'33.7"N, 100°29'41.7"E | MA |
| 15 | 23 February 2017 | *Malayemys subtrijuga* | Mueang Nonthaburi Market, Mueang, Nonthaburi | 13°50'33.7"N, 100°29'41.7"E | MA |
| 16 | 23 February 2017 | *Malayemys subtrijuga* | Mueang Nonthaburi Market, Mueang, Nonthaburi | 13°50'33.7"N, 100°29'41.7"E | MA |
| 17 | 23 February 2017 | *Malayemys subtrijuga* | Mueang Nonthaburi Market, Mueang, Nonthaburi | 13°50'33.7"N, 100°29'41.7"E | MA |
| 18 | 23 February 2017 | *Malayemys subtrijuga* | Mueang Nonthaburi Market, Mueang, Nonthaburi | 13°50'33.7"N, 100°29'41.7"E | MA |
| 19 | 23 February 2017 | *Malayemys subtrijuga* | Mueang Nonthaburi Market, Mueang, Nonthaburi | 13°50'33.7"N, 100°29'41.7"E | MA |
| 20 | 23 February 2017 | *Malayemys subtrijuga* | Mueang Nonthaburi Market, Mueang, Nonthaburi | 13°50'33.7"N, 100°29'41.7"E | MA |
| 21 | 23 February 2017 | *Malayemys subtrijuga* | Mueang Nonthaburi Market, Mueang, Nonthaburi | 13°50'33.7"N, 100°29'41.7"E | MA |
| 22 | 23 February 2017 | *Malayemys macrocephala* | Mueang Nonthaburi Market, Mueang, Nonthaburi | 13°50'33.7"N, 100°29'41.7"E | MA |
| 23 | 23 February 2017 | *Malayemys subtrijuga* | Mueang Nonthaburi Market, Mueang, Nonthaburi | 13°50'33.7"N, 100°29'41.7"E | MA |
| 24 | 23 February 2017 | *Malayemys subtrijuga* | Mueang Nonthaburi Market, Mueang, Nonthaburi | 13°50'33.7"N, 100°29'41.7"E | MA |
| 25 | 23 February 2017 | *Malayemys subtrijuga* | Mueang Nonthaburi Market, Mueang, Nonthaburi | 13°50'33.7"N, 100°29'41.7"E | MA |
| 26 | 23 February 2017 | *Malayemys macrocephala* | Mueang Nonthaburi Market, Mueang, Nonthaburi | 13°50'33.7"N, 100°29'41.7"E | MA |
| 27 | 23 February 2017 | *Malayemys subtrijuga* | Mueang Nonthaburi Market, Mueang, Nonthaburi | 13°50'33.7"N, 100°29'41.7"E | MA |
| 28 | 23 February 2017 | *Malayemys macrocephala* | Mueang Nonthaburi Market, Mueang, Nonthaburi | 13°50'33.7"N, 100°29'41.7"E | MA |
| 29 | 3 March 2017 | *Cuora amboinensis* | Prayasuren Temple, Khlong Sam Wa, Bangkok | 13°52'23.6"N, 100°42'03.6"E | CS |
| 30 | 3 March 2017 | *Cuora amboinensis* | Prayasuren Temple, Khlong Sam Wa, Bangkok | 13°52'23.6"N, 100°42'03.6"E | CS |
| 31 | 4 March 2017 | *Malayemys macrocephala* | Samma Chanyawat Temple, Bang Chan, Bangkok | 13°51'15.9"N, 100°41'37.6"E | CS |
| 32 | 4 March 2017 | *Malayemys macrocephala* | Samma Chanyawat Temple, Bang Chan, Bangkok | 13°51'15.9"N, 100°41'37.6"E | CS |
| 33 | 4 March 2017 | *Malayemys macrocephala* | Samma Chanyawat Temple, Bang Chan, Bangkok | 13°51'15.9"N, 100°41'37.6"E | CS |
| 34 | 4 March 2017 | *Malayemys subtrijuga* | Samma Chanyawat Temple, Bang Chan, Bangkok | 13°51'15.9"N, 100°41'37.6"E | CS |
| 35 | 4 March 2017 | *Malayemys macrocephala* | Samma Chanyawat Temple, Bang Chan, Bangkok | 13°51'15.9"N, 100°41'37.6"E | CS |
| 36 | 4 March 2017 | *Malayemys macrocephala* | Samma Chanyawat Temple, Bang Chan, Bangkok | 13°51'15.9"N, 100°41'37.6"E | CS |
| 37 | 4 March 2017 | *Malayemys subtrijuga* | Samma Chanyawat Temple, Bang Chan, Bangkok | 13°51'15.9"N, 100°41'37.6"E | CS |
| 38 | 4 March 2017 | *Malayemys subtrijuga* | Samma Chanyawat Temple, Bang Chan, Bangkok | 13°51'15.9"N, 100°41'37.6"E | CS |
| 39 | 4 March 2017 | *Malayemys macrocephala* | Samma Chanyawat Temple, Bang Chan, Bangkok | 13°51'15.9"N, 100°41'37.6"E | CS |
| 40 | 4 March 2017 | *Malayemys macrocephala* | Samma Chanyawat Temple, Bang Chan, Bangkok | 13°51'15.9"N, 100°41'37.6"E | CS |
| 41 | 4 March 2017 | *Malayemys macrocephala* | Samma Chanyawat Temple, Bang Chan, Bangkok | 13°51'15.9"N, 100°41'37.6"E | CS |
| 42 | 4 March 2017 | *Malayemys macrocephala* | Samma Chanyawat Temple, Bang Chan, Bangkok | 13°51'15.9"N, 100°41'37.6"E | CS |
| 43 | 15 March 2017 | *Malayemys subtrijuga* | Kasetsart University, Bangkhen, Bangkok | 13°50’53.6”N, 100°33’47.3”E | NH |
| 44 | 15 March 2017 | *Malayemys macrocephala* | Kasetsart University, Bangkhen, Bangkok | 13°50’53.6”N, 100°33’47.3”E | NH |
| 45 | 15 March 2017 | *Malayemys subtrijuga* | Kasetsart University, Bangkhen, Bangkok | 13°50’53.6”N, 100°33’47.3”E | NH |
| 46 | 15 March 2017 | *Malayemys macrocephala* | Kasetsart University, Bangkhen, Bangkok | 13°50’53.6”N, 100°33’47.3”E | NH |
| 47 | 15 March 2017 | *Malayemys macrocephala* | Kasetsart University, Bangkhen, Bangkok | 13°50’53.6”N, 100°33’47.3”E | NH |
| 48 | 17 March 2017 | *Trachemys scripta elegans* | Kasetsart University, Bangkhen, Bangkok | 13°50’53.6”N, 100°33’47.3”E | NH |
| 49 | 15 April 2017 | *Malayemys subtrijuga* | Kasetsart University, Bangkhen, Bangkok | 13°50’53.6”N, 100°33’47.3”E | NH |
| 50 | 15 April 2017 | *Malayemys macrocephala* | Kasetsart University, Bangkhen, Bangkok | 13°50’53.6”N, 100°33’47.3”E | NH |
| 51 | 15 April 2017 | *Malayemys macrocephala* | Kasetsart University, Bangkhen, Bangkok | 13°50’53.6”N, 100°33’47.3”E | NH |
| 52 | 15 April 2017 | *Malayemys subtrijuga* | Kasetsart University, Bangkhen, Bangkok | 13°50’53.6”N, 100°33’47.3”E | NH |
| 53 | 15 April 2017 | *Malayemys subtrijuga* | Kasetsart University, Bangkhen, Bangkok | 13°50’53.6”N, 100°33’47.3”E | NH |
| 54 | 17 April 2017 | *Malayemys macrocephala* | Warorot Market, Mueang, Chiangmai | 18°47'24.5"N, 99°00'01.8"E | MA |
| 55 | 17 April 2017 | *Malayemys macrocephala* | Warorot Market, Mueang, Chiangmai | 18°47'24.5"N, 99°00'01.8"E | MA |
| 56 | 17 April 2017 | *Malayemys macrocephala* | Warorot Market, Mueang, Chiangmai | 18°47'24.5"N, 99°00'01.8"E | MA |
| 57 | 17 April 2017 | *Malayemys macrocephala* | Warorot Market, Mueang, Chiangmai | 18°47'24.5"N, 99°00'01.8"E | MA |
| 58 | 17 April 2017 | *Malayemys macrocephala* | Warorot Market, Mueang, Chiangmai | 18°47'24.5"N, 99°00'01.8"E | MA |
| 59 | 15 May 2017 | *Malayemys macrocephala* | Kasetsart University, Bangkhen, Bangkok | 13°50’53.6”N, 100°33’47.3”E | NH |
| 60 | 15 May 2017 | *Malayemys macrocephala* | Kasetsart University, Bangkhen, Bangkok | 13°50’53.6”N, 100°33’47.3”E | NH |
| 61 | 15 May 2017 | *Malayemys macrocephala* | Kasetsart University, Bangkhen, Bangkok | 13°50’53.6”N, 100°33’47.3”E | NH |
| 62 | 15 May 2017 | *Malayemys macrocephala* | Kasetsart University, Bangkhen, Bangkok | 13°50’53.6”N, 100°33’47.3”E | NH |
| 63 | 15 May 2017 | *Malayemys macrocephala* | Kasetsart University, Bangkhen, Bangkok | 13°50’53.6”N, 100°33’47.3”E | NH |
| 64 | 15 June 2017 | *Malayemys subtrijuga* | Kasetsart University, Bangkhen, Bangkok | 13°50’53.6”N, 100°33’47.3”E | NH |
| 65 | 15 June 2017 | *Malayemys macrocephala* | Kasetsart University, Bangkhen, Bangkok | 13°50’53.6”N, 100°33’47.3”E | NH |
| 66 | 15 June 2017 | *Malayemys macrocephala* | Kasetsart University, Bangkhen, Bangkok | 13°50’53.6”N, 100°33’47.3”E | NH |
| 67 | 15 June 2017 | *Malayemys macrocephala* | Kasetsart University, Bangkhen, Bangkok | 13°50’53.6”N, 100°33’47.3”E | NH |
| 68 | 15 June 2017 | *Malayemys subtrijuga* | Kasetsart University, Bangkhen, Bangkok | 13°50’53.6”N, 100°33’47.3”E | NH |
| 69 | 1 July 2017 | *Siebenrockiella crassicollis* | Soi Thong Temple, Bang Sue, Bangkok | 13°81'21.0"N, 100°51'82.0"E | MA |
| 70 | 1 July 2017 | *Siebenrockiella crassicollis* | Soi Thong Temple, Bang Sue, Bangkok | 13°81'21.0"N, 100°51'82.0"E | MA |
| 71 | 1 July 2017 | *Siebenrockiella crassicollis* | Soi Thong Temple, Bang Sue, Bangkok | 13°81'21.0"N, 100°51'82.0"E | MA |
| 72 | 2 July 2017 | *Malayemys subtrijuga* | Chaiyo Worawihan Temple, Chai Yo, Ang Thong | 14°43'05.7"N, 100°26'18.6"E | CS |
| 73 | 2 July 2017 | *Malayemys macrocephala* | Chaiyo Worawihan Temple, Chai Yo, Ang Thong | 14°43'05.7"N, 100°26'18.6"E | CS |
| 74 | 8 July 2017 | *Malayemys macrocephala* | Lahan Temple, Si Prachan, Suphanburi | 14°60'44.1"N, 100°13'62.1"E | CS |
| 75 | 13 July 2017 | *Malayemys macrocephala* | Mahidol University, Phutthamonthon, Nakhon Pathom | 13°79'43.8"N, 100°32'73.2"E | NH |
| 76 | 15 July 2017 | *Malayemys subtrijuga* | Kasetsart University, Bangkhen, Bangkok | 13°50’53.6”N, 100°33’47.3”E | NH |
| 77 | 15 July 2017 | *Malayemys macrocephala* | Kasetsart University, Bangkhen, Bangkok | 13°50’53.6”N, 100°33’47.3”E | NH |
| 78 | 15 July 2017 | *Malayemys subtrijuga* | Kasetsart University, Bangkhen, Bangkok | 13°50’53.6”N, 100°33’47.3”E | NH |
| 79 | 15 July 2017 | *Malayemys macrocephala* | Kasetsart University, Bangkhen, Bangkok | 13°50’53.6”N, 100°33’47.3”E | NH |
| 80 | 15 July 2017 | *Malayemys subtrijuga* | Kasetsart University, Bangkhen, Bangkok | 13°50’53.6”N, 100°33’47.3”E | NH |
| 81 | 19 July 2017 | *Hieremys annandalii* | Kasetsart University, Bangkhen, Bangkok | 13°50’53.6”N, 100°33’47.3”E | NH |
| 82 | 15 August 2017 | *Malayemys macrocephala* | Kasetsart University, Bangkhen, Bangkok | 13°50’53.6”N, 100°33’47.3”E | NH |
| 83 | 15 August 2017 | *Malayemys subtrijuga* | Kasetsart University, Bangkhen, Bangkok | 13°50’53.6”N, 100°33’47.3”E | NH |
| 84 | 15 August 2017 | *Malayemys macrocephala* | Kasetsart University, Bangkhen, Bangkok | 13°50’53.6”N, 100°33’47.3”E | NH |
| 85 | 15 August 2017 | *Malayemys subtrijuga* | Kasetsart University, Bangkhen, Bangkok | 13°50’53.6”N, 100°33’47.3”E | NH |
| 86 | 15 August 2017 | *Malayemys subtrijuga* | Kasetsart University, Bangkhen, Bangkok | 13°50’53.6”N, 100°33’47.3”E | NH |
| 87 | 17 August 2017 | *Cyclemys oldhamii* | Doi Muser Market, Mae Sot, Tak | 16°45'43.6"N, 98°54'21.5"E | MA |
| 88 | 15 September 2017 | *Malayemys subtrijuga* | Kasetsart University, Bangkhen, Bangkok | 13°50’53.6”N, 100°33’47.3”E | NH |
| 89 | 15 September 2017 | *Malayemys subtrijuga* | Kasetsart University, Bangkhen, Bangkok | 13°50’53.6”N, 100°33’47.3”E | NH |
| 90 | 15 September 2017 | *Malayemys subtrijuga* | Kasetsart University, Bangkhen, Bangkok | 13°50’53.6”N, 100°33’47.3”E | NH |
| 91 | 15 September 2017 | *Malayemys macrocephala* | Kasetsart University, Bangkhen, Bangkok | 13°50’53.6”N, 100°33’47.3”E | NH |
| 92 | 15 September 2017 | *Malayemys subtrijuga* | Kasetsart University, Bangkhen, Bangkok | 13°50’53.6”N, 100°33’47.3”E | NH |
| 93 | 30 September 2017 | *Malayemys subtrijuga* | Bang Yai Market, Bang Yai, Nonthaburi | 13°87'79.9"N, 100°40'68.4"E | MA |
| 94 | 30 September 2017 | *Malayemys subtrijuga* | Bang Yai Market, Bang Yai, Nonthaburi | 13°87'79.9"N, 100°40'68.4"E | MA |
| 95 | 30 September 2017 | *Malayemys subtrijuga* | Bang Yai Market, Bang Yai, Nonthaburi | 13°87'79.9"N, 100°40'68.4"E | MA |
| 96 | 30 September 2017 | *Malayemys subtrijuga* | Bang Yai Market, Bang Yai, Nonthaburi | 13°87'79.9"N, 100°40'68.4"E | MA |
| 97 | 30 September 2017 | *Malayemys subtrijuga* | Bang Yai Market, Bang Yai, Nonthaburi | 13°87'79.9"N, 100°40'68.4"E | MA |
| 98 | 30 September 2017 | *Malayemys subtrijuga* | Bang Yai Market, Bang Yai, Nonthaburi | 13°87'79.9"N, 100°40'68.4"E | MA |
| 99 | 30 September 2017 | *Malayemys subtrijuga* | Bang Yai Market, Bang Yai, Nonthaburi | 13°87'79.9"N, 100°40'68.4"E | MA |
| 100 | 30 September 2017 | *Malayemys macrocephala* | Bang Yai Market, Bang Yai, Nonthaburi | 13°87'79.9"N, 100°40'68.4"E | MA |
| 101 | 30 September 2017 | *Malayemys subtrijuga* | Bang Yai Market, Bang Yai, Nonthaburi | 13°87'79.9"N, 100°40'68.4"E | MA |
| 101 | 30 September 2017 | *Malayemys subtrijuga* | Bang Yai Market, Bang Yai, Nonthaburi | 13°87'79.9"N, 100°40'68.4"E | MA |
| 103 | 30 September 2017 | *Malayemys subtrijuga* | Bang Yai Market, Bang Yai, Nonthaburi | 13°87'79.9"N, 100°40'68.4"E | MA |
| 104 | 30 September 2017 | *Malayemys subtrijuga* | Bang Yai Market, Bang Yai, Nonthaburi | 13°87'79.9"N, 100°40'68.4"E | MA |
| 105 | 1 October 2017 | *Malayemys subtrijuga* | Wiset Chai Chan, Ang Thong | 14°36'59.3"N, 100°23'24.6"E | NH |
| 106 | 1 October 2017 | *Malayemys subtrijuga* | Wiset Chai Chan, Ang Thong | 14°36'59.3"N, 100°23'24.6"E | NH |
| 107 | 1 October 2017 | *Malayemys subtrijuga* | Wiset Chai Chan, Ang Thong | 14°36'59.3"N, 100°23'24.6"E | NH |
| 108 | 1 October 2017 | *Malayemys subtrijuga* | Wiset Chai Chan, Ang Thong | 14°36'59.3"N, 100°23'24.6"E | NH |
| 109 | 3 October 2017 | *Malayemys subtrijuga* | Lahan Temple, Si Prachan, Suphanburi | 14°60'44.1"N, 100°13'62.1"E | CS |
| 110 | 3 October 2017 | *Malayemys macrocephala* | Lahan Temple, Si Prachan, Suphanburi | 14°60'44.1"N, 100°13'62.1"E | CS |
| 111 | 3 October 2017 | *Malayemys subtrijuga* | Lahan Temple, Si Prachan, Suphanburi | 14°60'44.1"N, 100°13'62.1"E | CS |
| 112 | 3 October 2017 | *Malayemys subtrijuga* | Lahan Temple, Si Prachan, Suphanburi | 14°60'44.1"N, 100°13'62.1"E | CS |
| 113 | 15 October 2017 | *Malayemys subtrijuga* | Kasetsart University, Bangkhen, Bangkok | 13°50’53.6”N, 100°33’47.3”E | NH |
| 114 | 15 October 2017 | *Malayemys subtrijuga* | Kasetsart University, Bangkhen, Bangkok | 13°50’53.6”N, 100°33’47.3”E | NH |
| 115 | 15 October 2017 | *Malayemys macrocephala* | Kasetsart University, Bangkhen, Bangkok | 13°50’53.6”N, 100°33’47.3”E | NH |
| 116 | 15 October 2017 | *Malayemys subtrijuga* | Kasetsart University, Bangkhen, Bangkok | 13°50’53.6”N, 100°33’47.3”E | NH |
| 117 | 15 October 2017 | *Malayemys macrocephala* | Kasetsart University, Bangkhen, Bangkok | 13°50’53.6”N, 100°33’47.3”E | NH |
| 118 | 24 October 2017 | *Hieremys annandalii* | Dhamma Wutharam Temple, Kapur, Ranong | 9°35'07.5"N 98°36'08.6"E | CS |
| 119 | 24 October 2017 | *Hieremys annandalii* | Dhamma Wutharam Temple, Kapur, Ranong | 9°35'07.5"N 98°36'08.6"E | CS |
| 120 | 24 October 2017 | *Hieremys annandalii* | Dhamma Wutharam Temple, Kapur, Ranong | 9°35'07.5"N 98°36'08.6"E | CS |
| 121 | 24 October 2017 | *Cuora amboinensis* | Dhamma Wutharam Temple, Kapur, Ranong | 9°35'07.5"N 98°36'08.6"E | CS |
| 122 | 24 October 2017 | *Cuora amboinensis* | Dhamma Wutharam Temple, Kapur, Ranong | 9°35'07.5"N 98°36'08.6"E | CS |
| 123 | 24 October 2017 | *Cuora amboinensis* | Dhamma Wutharam Temple, Kapur, Ranong | 9°35'07.5"N 98°36'08.6"E | CS |
| 124 | 24 October 2017 | *Cuora amboinensis* | Dhamma Wutharam Temple, Kapur, Ranong | 9°35'07.5"N 98°36'08.6"E | CS |
| 125 | 24 October 2017 | *Cuora amboinensis* | Dhamma Wutharam Temple, Kapur, Ranong | 9°35'07.5"N 98°36'08.6"E | CS |
| 126 | 24 October 2017 | *Cuora amboinensis* | Dhamma Wutharam Temple, Kapur, Ranong | 9°35'07.5"N 98°36'08.6"E | CS |
| 127 | 24 October 2017 | *Cuora amboinensis* | Dhamma Wutharam Temple, Kapur, Ranong | 9°35'07.5"N 98°36'08.6"E | CS |
| 128 | 24 October 2017 | *Cuora amboinensis* | Dhamma Wutharam Temple, Kapur, Ranong | 9°35'07.5"N 98°36'08.6"E | CS |
| 129 | 24 October 2017 | *Cuora amboinensis* | Dhamma Wutharam Temple, Kapur, Ranong | 9°35'07.5"N 98°36'08.6"E | CS |
| 130 | 24 October 2017 | *Cuora amboinensis* | Dhamma Wutharam Temple, Kapur, Ranong | 9°35'07.5"N 98°36'08.6"E | CS |
| 131 | 24 October 2017 | *Cuora amboinensis* | Dhamma Wutharam Temple, Kapur, Ranong | 9°35'07.5"N 98°36'08.6"E | CS |
| 132 | 24 October 2017 | *Cuora amboinensis* | Dhamma Wutharam Temple, Kapur, Ranong | 9°35'07.5"N 98°36'08.6"E | CS |
| 133 | 24 October 2017 | *Cuora amboinensis* | Dhamma Wutharam Temple, Kapur, Ranong | 9°35'07.5"N 98°36'08.6"E | CS |
| 134 | 24 October 2017 | *Cuora amboinensis* | Dhamma Wutharam Temple, Kapur, Ranong | 9°35'07.5"N 98°36'08.6"E | CS |
| 135 | 24 October 2017 | *Cuora amboinensis* | Dhamma Wutharam Temple, Kapur, Ranong | 9°35'07.5"N 98°36'08.6"E | CS |
| 136 | 24 October 2017 | *Cuora amboinensis* | Dhamma Wutharam Temple, Kapur, Ranong | 9°35'07.5"N 98°36'08.6"E | CS |
| 137 | 24 October 2017 | *Cuora amboinensis* | Dhamma Wutharam Temple, Kapur, Ranong | 9°35'07.5"N 98°36'08.6"E | CS |
| 138 | 24 October 2017 | *Cuora amboinensis* | Dhamma Wutharam Temple, Kapur, Ranong | 9°35'07.5"N 98°36'08.6"E | CS |
| 139 | 24 October 2017 | *Cuora amboinensis* | Dhamma Wutharam Temple, Kapur, Ranong | 9°35'07.5"N 98°36'08.6"E | CS |
| 140 | 24 October 2017 | *Cuora amboinensis* | Dhamma Wutharam Temple, Kapur, Ranong | 9°35'07.5"N 98°36'08.6"E | CS |
| 141 | 11 November 2017 | *Cyclemys oldhamii* | Bang Saphan Noi, Prachuap Khiri Khan | 11°02'02.6"N, 99°14'25.3"E | NH |
| 142 | 15 November 2017 | *Malayemys macrocephala* | Kasetsart University, Bangkhen, Bangkok | 13°50’53.6”N, 100°33’47.3”E | NH |
| 143 | 15 November 2017 | *Malayemys subtrijuga* | Kasetsart University, Bangkhen, Bangkok | 13°50’53.6”N, 100°33’47.3”E | NH |
| 144 | 15 November 2017 | *Malayemys macrocephala* | Kasetsart University, Bangkhen, Bangkok | 13°50’53.6”N, 100°33’47.3”E | NH |
| 145 | 15 November 2017 | *Malayemys macrocephala* | Kasetsart University, Bangkhen, Bangkok | 13°50’53.6”N, 100°33’47.3”E | NH |
| 146 | 15 November 2017 | *Malayemys subtrijuga* | Kasetsart University, Bangkhen, Bangkok | 13°50’53.6”N, 100°33’47.3”E | NH |
| 147 | 11 November 2017 | *Hieremys annandalii* | Thong Pha Phum, Kanchanaburi | 14°35'46.2"N, 98°43'42.1"E | MA |
| 148 | 11 November 2017 | *Cuora amboinensis* | Thong Pha Phum, Kanchanaburi | 14°35'46.2"N, 98°43'42.1"E | MA |
| 149 | 15 December 2017 | *Malayemys macrocephala* | Kasetsart University, Bangkhen, Bangkok | 13°50’53.6”N, 100°33’47.3”E | NH |
| 150 | 15 December 2017 | *Malayemys subtrijuga* | Kasetsart University, Bangkhen, Bangkok | 13°50’53.6”N, 100°33’47.3”E | NH |
| 151 | 15 December 2017 | *Malayemys subtrijuga* | Kasetsart University, Bangkhen, Bangkok | 13°50’53.6”N, 100°33’47.3”E | NH |
| 152 | 15 December 2017 | *Malayemys macrocephala* | Kasetsart University, Bangkhen, Bangkok | 13°50’53.6”N, 100°33’47.3”E | NH |
| 153 | 15 December 2017 | *Malayemys subtrijuga* | Kasetsart University, Bangkhen, Bangkok | 13°50’53.6”N, 100°33’47.3”E | NH |
| 154 | 15 January 2018 | *Malayemys subtrijuga* | Kasetsart University, Bangkhen, Bangkok | 13°50’53.6”N, 100°33’47.3”E | NH |
| 155 | 15 January 2018 | *Malayemys subtrijuga* | Kasetsart University, Bangkhen, Bangkok | 13°50’53.6”N, 100°33’47.3”E | NH |
| 156 | 15 January 2018 | *Malayemys macrocephala* | Kasetsart University, Bangkhen, Bangkok | 13°50’53.6”N, 100°33’47.3”E | NH |
| 157 | 15 January 2018 | *Malayemys subtrijuga* | Kasetsart University, Bangkhen, Bangkok | 13°50’53.6”N, 100°33’47.3”E | NH |
| 158 | 15 January 2018 | *Malayemys subtrijuga* | Kasetsart University, Bangkhen, Bangkok | 13°50’53.6”N, 100°33’47.3”E | NH |
| 159 | 15 January 2018 | *Trachemys scripta elegans* | Kasetsart University, Bangkhen, Bangkok | 13°50’53.6”N, 100°33’47.3”E | NH |
| 160 | 15 January 2018 | *Trachemys scripta elegans* | Kasetsart University, Bangkhen, Bangkok | 13°50’53.6”N, 100°33’47.3”E | NH |
| 161 | 14 April 2018 | *Malayemys subtrijuga* | Phikul Kaew Temple, Ban Na, Nakhon Nayok | 14°15'13.7"N, 101°03'53.4"E | MA |
| 162 | 14 April 2018 | *Malayemys subtrijuga* | Phikul Kaew Temple, Ban Na, Nakhon Nayok | 14°15'13.7"N, 101°03'53.4"E | MA |
| 163 | 14 April 2018 | *Malayemys subtrijuga* | Phikul Kaew Temple, Ban Na, Nakhon Nayok | 14°15'13.7"N, 101°03'53.4"E | MA |
| 164 | 14 April 2018 | *Malayemys subtrijuga* | Phikul Kaew Temple, Ban Na, Nakhon Nayok | 14°15'13.7"N, 101°03'53.4"E | MA |
| 165 | 14 April 2018 | *Malayemys subtrijuga* | Phikul Kaew Temple, Ban Na, Nakhon Nayok | 14°15'13.7"N, 101°03'53.4"E | MA |
| 166 | 14 April 2018 | *Malayemys subtrijuga* | Phikul Kaew Temple, Ban Na, Nakhon Nayok | 14°15'13.7"N, 101°03'53.4"E | MA |
| 167 | 14 April 2018 | *Malayemys subtrijuga* | Phikul Kaew Temple, Ban Na, Nakhon Nayok | 14°15'13.7"N, 101°03'53.4"E | MA |
| 168 | 18 April 2018 | *Hieremys annandalii* | Thepnorarat Temple, Mueang, Samut Sakhon | 13°35'03.3"N, 100°17'36.7"E | CS |
| 169 | 18 April 2018 | *Hieremys annandalii* | Thepnorarat Temple, Mueang, Samut Sakhon | 13°35'03.3"N, 100°17'36.7"E | CS |
| 170 | 20 April 2018 | *Malayemys macrocephala* | Lam Luk Ka, Pathum Thani | 13°55'55.2"N, 100°43'04.9"E | NH |
| 171 | 20 April 2018 | *Malayemys macrocephala* | Lam Luk Ka, Pathum Thani | 13°55'55.2"N, 100°43'04.9"E | NH |
| 172 | 20 April 2018 | *Malayemys macrocephala* | Lam Luk Ka, Pathum Thani | 13°55'55.2"N, 100°43'04.9"E | NH |
| 173 | 20 May 2018 | *Hieremys annandalii* | Sammachanyawat Temple, Khlong Sam Wa, Bangkok | 13°51'16.0"N, 100°41'36.8"E | CS |
| 174 | 20 May 2018 | *Hieremys annandalii* | Sammachanyawat Temple, Khlong Sam Wa, Bangkok | 13°51'16.0"N, 100°41'36.8"E | CS |
| 175 | 20 May 2018 | *Hieremys annandalii* | Sammachanyawat Temple, Khlong Sam Wa, Bangkok | 13°51'16.0"N, 100°41'36.8"E | CS |
| 176 | 26 May 2018 | *Hieremys annandalii* | Buddhist Association Ban Thunghiang, Phanat Nikhom, Chonburi | 13°21'25.4"N, 101°13'41.3"E | CS |
| 177 | 26 May 2018 | *Hieremys annandalii* | Buddhist Association Ban Thunghiang, Phanat Nikhom, Chonburi | 13°21'25.4"N, 101°13'41.3"E | CS |
| 178 | 26 May 2018 | *Hieremys annandalii* | Buddhist Association Ban Thunghiang, Phanat Nikhom, Chonburi | 13°21'25.4"N, 101°13'41.3"E | CS |
| 179 | 26 May 2018 | *Hieremys annandalii* | Buddhist Association Ban Thunghiang, Phanat Nikhom, Chonburi | 13°21'25.4"N, 101°13'41.3"E | CS |
| 180 | 26 May 2018 | *Hieremys annandalii* | Buddhist Association Ban Thunghiang, Phanat Nikhom, Chonburi | 13°21'25.4"N, 101°13'41.3"E | CS |
| 181 | 26 May 2018 | *Hieremys annandalii* | Buddhist Association Ban Thunghiang, Phanat Nikhom, Chonburi | 13°21'25.4"N, 101°13'41.3"E | CS |
| 182 | 26 May 2018 | *Hieremys annandalii* | Buddhist Association Ban Thunghiang, Phanat Nikhom, Chonburi | 13°21'25.4"N, 101°13'41.3"E | CS |
| 183 | 26 May 2018 | *Hieremys annandalii* | Buddhist Association Ban Thunghiang, Phanat Nikhom, Chonburi | 13°21'25.4"N, 101°13'41.3"E | CS |
| 184 | 26 May 2018 | *Hieremys annandalii* | Buddhist Association Ban Thunghiang, Phanat Nikhom, Chonburi | 13°21'25.4"N, 101°13'41.3"E | CS |
| 185 | 26 May 2018 | *Hieremys annandalii* | Buddhist Association Ban Thunghiang, Phanat Nikhom, Chonburi | 13°21'25.4"N, 101°13'41.3"E | CS |
| 186 | 26 May 2018 | *Hieremys annandalii* | Buddhist Association Ban Thunghiang, Phanat Nikhom, Chonburi | 13°21'25.4"N, 101°13'41.3"E | CS |
| 187 | 26 May 2018 | *Hieremys annandalii* | Buddhist Association Ban Thunghiang, Phanat Nikhom, Chonburi | 13°21'25.4"N, 101°13'41.3"E | CS |
| 188 | 26 May 2018 | *Cuora amboinensis* | Buddhist Association Ban Thunghiang, Phanat Nikhom, Chonburi | 13°21'25.4"N, 101°13'41.3"E | CS |
| 189 | 26 May 2018 | *Cuora amboinensis* | Buddhist Association Ban Thunghiang, Phanat Nikhom, Chonburi | 13°21'25.4"N, 101°13'41.3"E | CS |
| 190 | 26 May 2018 | *Cuora amboinensis* | Buddhist Association Ban Thunghiang, Phanat Nikhom, Chonburi | 13°21'25.4"N, 101°13'41.3"E | CS |
| 191 | 26 May 2018 | *Cuora amboinensis* | Buddhist Association Ban Thunghiang, Phanat Nikhom, Chonburi | 13°21'25.4"N, 101°13'41.3"E | CS |
| 192 | 26 May 2018 | *Cuora amboinensis* | Buddhist Association Ban Thunghiang, Phanat Nikhom, Chonburi | 13°21'25.4"N, 101°13'41.3"E | CS |
| 193 | 26 May 2018 | *Cuora amboinensis* | Buddhist Association Ban Thunghiang, Phanat Nikhom, Chonburi | 13°21'25.4"N, 101°13'41.3"E | CS |
| 194 | 26 May 2018 | *Cuora amboinensis* | Buddhist Association Ban Thunghiang, Phanat Nikhom, Chonburi | 13°21'25.4"N, 101°13'41.3"E | CS |
| 195 | 26 May 2018 | *Cuora amboinensis* | Buddhist Association Ban Thunghiang, Phanat Nikhom, Chonburi | 13°21'25.4"N, 101°13'41.3"E | CS |
| 196 | 26 May 2018 | *Cuora amboinensis* | Buddhist Association Ban Thunghiang, Phanat Nikhom, Chonburi | 13°21'25.4"N, 101°13'41.3"E | CS |
| 197 | 26 May 2018 | *Cuora amboinensis* | Buddhist Association Ban Thunghiang, Phanat Nikhom, Chonburi | 13°21'25.4"N, 101°13'41.3"E | CS |
| 198 | 26 May 2018 | *Cuora amboinensis* | Buddhist Association Ban Thunghiang, Phanat Nikhom, Chonburi | 13°21'25.4"N, 101°13'41.3"E | CS |
| 199 | 26 May 2018 | *Cuora amboinensis* | Buddhist Association Ban Thunghiang, Phanat Nikhom, Chonburi | 13°21'25.4"N, 101°13'41.3"E | CS |
| 200 | 26 May 2018 | *Cuora amboinensis* | Buddhist Association Ban Thunghiang, Phanat Nikhom, Chonburi | 13°21'25.4"N, 101°13'41.3"E | CS |
| 201 | 26 May 2018 | *Cuora amboinensis* | Buddhist Association Ban Thunghiang, Phanat Nikhom, Chonburi | 13°21'25.4"N, 101°13'41.3"E | CS |
| 202 | 26 May 2018 | *Cuora amboinensis* | Buddhist Association Ban Thunghiang, Phanat Nikhom, Chonburi | 13°21'25.4"N, 101°13'41.3"E | CS |
| 203 | 26 May 2018 | *Cuora amboinensis* | Buddhist Association Ban Thunghiang, Phanat Nikhom, Chonburi | 13°21'25.4"N, 101°13'41.3"E | CS |
| 204 | 26 May 2018 | *Cuora amboinensis* | Buddhist Association Ban Thunghiang, Phanat Nikhom, Chonburi | 13°21'25.4"N, 101°13'41.3"E | CS |
| 205 | 26 May 2018 | *Cuora amboinensis* | Buddhist Association Ban Thunghiang, Phanat Nikhom, Chonburi | 13°21'25.4"N, 101°13'41.3"E | CS |
| 206 | 26 May 2018 | *Cuora amboinensis* | Buddhist Association Ban Thunghiang, Phanat Nikhom, Chonburi | 13°21'25.4"N, 101°13'41.3"E | CS |
| 207 | 26 May 2018 | *Cuora amboinensis* | Buddhist Association Ban Thunghiang, Phanat Nikhom, Chonburi | 13°21'25.4"N, 101°13'41.3"E | CS |
| 208 | 26 May 2018 | *Cuora amboinensis* | Buddhist Association Ban Thunghiang, Phanat Nikhom, Chonburi | 13°21'25.4"N, 101°13'41.3"E | CS |
| 209 | 26 May 2018 | *Cuora amboinensis* | Buddhist Association Ban Thunghiang, Phanat Nikhom, Chonburi | 13°21'25.4"N, 101°13'41.3"E | CS |
| 210 | 26 May 2018 | *Cuora amboinensis* | Buddhist Association Ban Thunghiang, Phanat Nikhom, Chonburi | 13°21'25.4"N, 101°13'41.3"E | CS |
| 211 | 26 May 2018 | *Heosemys grandis* | Buddhist Association Ban Thunghiang, Phanat Nikhom, Chonburi | 13°21'25.4"N, 101°13'41.3"E | CS |
| 212 | 26 May 2018 | *Heosemys grandis* | Buddhist Association Ban Thunghiang, Phanat Nikhom, Chonburi | 13°21'25.4"N, 101°13'41.3"E | CS |
| 213 | 26 May 2018 | *Heosemys grandis* | Buddhist Association Ban Thunghiang, Phanat Nikhom, Chonburi | 13°21'25.4"N, 101°13'41.3"E | CS |
| 214 | 26 May 2018 | *Trachemys scripta elegans* | Buddhist Association Ban Thunghiang, Phanat Nikhom, Chonburi | 13°21'25.4"N, 101°13'41.3"E | CS |
| 215 | 5 June 2018 | *Cuora amboinensis* | Sadao, Songkhla | 6°37'27.7"N, 100°24'41.4"E | NH |
| 216 | 5 June 2018 | *Cuora amboinensis* | Sadao, Songkhla | 6°37'27.7"N, 100°24'41.4"E | NH |
| 217 | 5 June 2018 | *Cuora amboinensis* | Sadao, Songkhla | 6°37'27.7"N, 100°24'41.4"E | NH |
| 218 | 5 June 2018 | *Cuora amboinensis* | Sadao, Songkhla | 6°37'27.7"N, 100°24'41.4"E | NH |
| 219 | 5 June 2018 | *Cuora amboinensis* | Sadao, Songkhla | 6°37'27.7"N, 100°24'41.4"E | NH |
| 220 | 5 June 2018 | *Cuora amboinensis* | Sadao, Songkhla | 6°37'27.7"N, 100°24'41.4"E | NH |
| 221 | 5 June 2018 | *Cuora amboinensis* | Sadao, Songkhla | 6°37'27.7"N, 100°24'41.4"E | NH |
| 222 | 28 June 2018 | *Malayemys khoratensis* | Mueang, Udon Thani | 17°25'02.8"N 102°46'52.4"E | NH |

|  |  | |  |  |
| --- | --- | --- | --- | --- |
|  |  |  |  |  |
|  |  |  |  |  |
|  |  |  |  |  |
|  |  |  |  |  |
|  |  |  |  |  |
|  |  |  |  |  |
|  |  |  |  |  |
|  |  |  |  |  |
|  |  |  |  |  |
|  |  |  |  |  |
